# Optimal value of CA19-9 determined by *KRAS*-mutated circulating tumor DNA contributes to the prediction of prognosis in pancreatic cancer patients

**DOI:** 10.1038/s41598-021-00060-9

**Published:** 2021-10-21

**Authors:** Fumiaki Watanabe, Koichi Suzuki, Sawako Tamaki, Iku Abe, Yuhei Endo, Yuji Takayama, Hideki Ishikawa, Nao Kakizawa, Masaaki Saito, Kazushige Futsuhara, Hiroshi Noda, Fumio Konishi, Toshiki Rikiyama

**Affiliations:** 1 Department of Surgery, Saitama Medical Center, Jichi Medical University, 1-847, Amanuma-cho, Omiya-ku, Saitama 330-8503 Japan; 2 Nerima Hikarigaoka Hospital, 2-11-1, Hikarigaoka, Nerima-ku, Tokyo 179-0072 Japan

**Keywords:** Cancer, Gastroenterology, Medical research, Oncology, Risk factors

## Abstract

Despite the acceptance of carbohydrate antigen 19-9 (CA19-9) as a valuable predictor for the prognosis of pancreatic ductal adenocarcinoma (PDAC), its cutoff value remains controversial. Our previous study showed a significant correlation between CA19-9 levels and the presence of *KRAS*-mutated ctDNA in the blood of patients with PDAC. Based on this correlation, we investigated the optimal cutoff value of CA19-9 before surgery. Continuous CA19-9 values and *KRAS*-mutated ctDNAs were monitored in 22 patients with unresectable PDAC who underwent chemotherapy between 2015 and 2017. Receiver operating characteristic curve analysis identified 949.7 U/mL of CA19-9 as the cutoff value corresponding to the presence of *KRAS*-mutated ctDNA. The median value of CA19-9 was 221.1 U/mL. Subsequently, these values were verified for their prognostic values of recurrence-free survival (RFS) and overall survival (OS) in 60 patients who underwent surgery between 2005 and 2013. Multivariate analysis revealed that 949.7 U/mL of CA19-9 was an independent risk factor for OS and RFS in these patients (*P* = 0.001 and *P* = 0.010, respectively), along with lymph node metastasis (*P* = 0.008 and *P* = 0.017), unlike the median CA19-9 level (*P* = 0.150 and *P* = 0.210). The optimal CA19-9 level contributes to the prediction of prognosis in patients with PDAC before surgery.

## Introduction

Pancreatic ductal adenocarcinoma (PDAC) is the seventh leading cause of cancer mortality and the twelfth most common malignancy worldwide^1^. Based on the expected demographic shift, PDAC will become the second leading cause of cancer-related deaths by 2030^2^. Recently, after resection and chemotherapy, the median survival time has improved to about 30 months and the 5-year survival rate to about 30% when combined with modern combination chemotherapy; however, this is still not satisfactory^3^. The most effective treatment should be determined according to the survival benefit of each patient. Individualization of cancer therapy is a future perspective to improve patient prognosis.

Various genetic and molecular alterations have been identified in pancreatic cancer, including mutations in *KRAS*, *p16*, *p53*, *BRCA2*, *Smad4*, and other alterations^4^. However, the translation of this scientific knowledge into clinical treatment regimens is still largely unrealized. Tumor marker-adjusted surgical and nonsurgical therapy for pancreatic cancer has been discussed by several authors^5–10^. At present, carbohydrate antigen 19-9 (CA19-9) may be the most appropriate for this purpose because of its secretion in approximately 75–80% of pancreatic cancer patients. The levels of CA19-9 correlate with tumor size, stage, and burden^11^. Therefore, CA19-9 has been commonly used to establish the diagnosis, assess resectability, monitor progression, and determine the prognosis of PDAC^12^. Pre- and postoperative CA19-9 levels may even predict prognosis^13–17^.

Despite the acceptance of CA19-9 as a valuable predictor for the prognosis of PDAC, its usefulness remains controversial^12,18,19^. Similarly, elevated levels of CA19-9 are observed in many benign illnesses, such as liver disease, cholangitis, and pancreatitis, and are not applicable in patients with the Lewis antigen-negative blood group^20^. Additionally, hepatic and pancreatic cysts may also interfere with CA19-9 levels^21,22^.

As an alternative to CA19-9, detection of circulating tumor DNA (ctDNA) extracted from the plasma and other body fluids, known as liquid biopsy, is a promising tool for molecular diagnostics of cancer patients^23–27^. Solid tumors, including colorectal cancer, breast cancer, and PDAC, discharge DNA fragments into systemic circulation^28^. Since liquid biopsy is an ideal noninvasive tool that allows multiple testing over time, tumor dynamics can be observed by longitudinal monitoring of mutated ctDNA^29^. Our previous studies showed that longitudinal monitoring of mutated ctDNA indicated tumor dynamics regarding various treatments for patients with colorectal and pancreatic cancer, which in turn provided useful information for treatment determination^30,31^. In addition, we showed a significant correlation between *KRAS*-mutated ctDNA and the value of CA19-9 in PDAC^31^.

Based on this evidence regarding the relationship between *KRAS*-mutated ctDNA and CA19-9, we aimed to investigate the optimal cutoff value of CA19-9 according to the presence of *KRAS*-mutated ctDNA, determined by liquid biopsy, in patients with unresectable PDAC. Additionally, we explored the clinical relevance of this modified cutoff value of CA19-9 in patients with resectable PDAC to predict recurrence and prognosis.

## Results

### Characteristics of patients who underwent chemotherapy

Table 1 shows the characteristics of patients who underwent chemotherapy. In total, 22 patients with locally advanced (n = 8; 36.4%) and metastatic (n = 14; 63.6%) PDAC were included in this study. Patients underwent chemotherapy with FOLFIRINOX (n = 5; 22.7%), gemcitabine plus nab-paclitaxel (n = 16; 72.7%), or gemcitabine only (n = 1; 4.6%) as first-line drugs. CA19-9 and *KRAS*-mutated ctDNAs were monitored to assess drug response. Prior to the investigation of *KRAS-*mutated ctDNA in plasma, *KRAS* assessment was performed in tumor tissues of 22 patients with PDAC who underwent chemotherapy using RASKET with a sensitivity of 1–5% and droplet digital polymerase chain reaction (ddPCR) with a sensitivity of 0.01–0.1%. Regarding frequency, G12D, G12V, G12V + G12D, G12R + G12D, Q61H, and Q61H + G12D were detected in six (27.3%), five (22.7%), four (18.2%), three (13.6%), one (4.5%), and one (4.5%) of the 22 samples, respectively.

**Table 1:** Characteristics of patients who underwent chemotherapy.

Characteristics	Value (N = 22)
**Sex**	
Male	10 (45.5%)
Female	12 (54.5%)
**Age (median, 69 years)**	
> 70 years	9 (40.9%)
≤ 70 years	13 (59.1%)
**UICC stage**	
Stage III	8 (36.4%)
Stage IV	14 (63.6%)
**CA19-9 level before treatment**	
≥ 37 U/mL	18 (81.8%)
< 37 U/mL	4 (18.2%)
**First-line chemotherapy regimen**	
FOLFIRINOX	5 (22.7%)
Gemcitabine + nab-paclitaxel	16 (72.7%)
Gemcitabine	1 (4.6%)
*KRAS* status in tumor tissue	
12D	6 (27.3%)
12V	5 (22.7%)
12 V, 12D	4 (18.2%)
12R, 12D	3 (13.6%)
Q61H	1 (4.5%)
Q61H, 12D	1 (4.5%)
ND	2 (9.1%)

Data are presented as n (%). UICC, Union for International Cancer Control; CA19-9, carbohydrate antigen 19-9; FOLFIRINOX, folinic acid, fluorouracil, irinotecan, and oxaliplatin; ND, not determined.

### Sequential assessments of ctDNA and CA19-9 in patients who underwent chemotherapy

**Figure 1:**
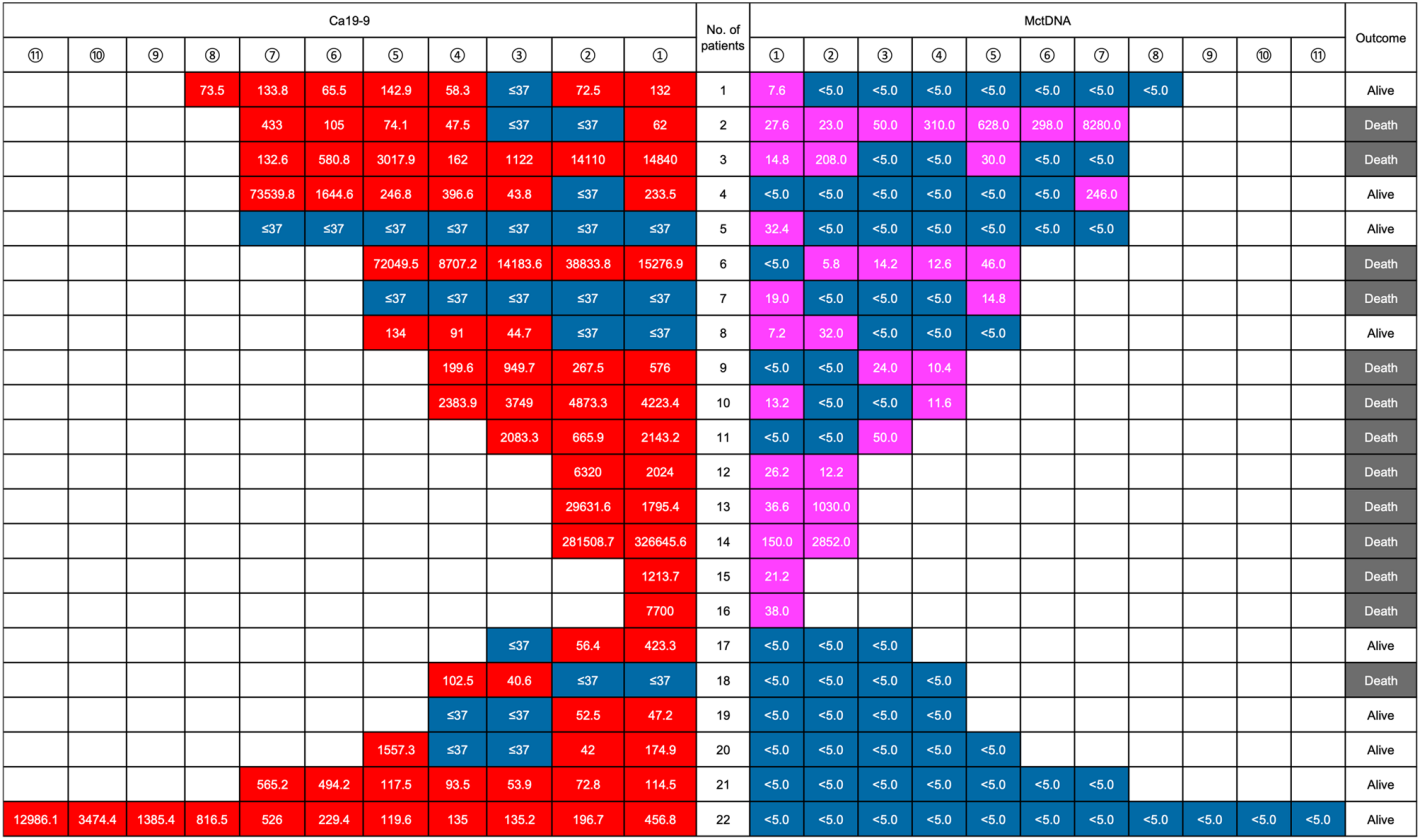
Sequential assessments of *KRAS*-mutated ctDNA and carbohydrate antigen 19-9 (CA19-9) value in longitudinal monitoring. CA19-9 levels and the emergence of *KRAS*-mutated ctDNA are shown under “CA19-9” and “MctDNA”, respectively, and are ordered as per the timing of blood examination during chemotherapy (1 → 11). CA19-9< 7 U/mL and no detection of *KRAS*-mutated ctDNA are represented in blue, whereas CA19-9≥37 U/mL and the emergence of *KRAS*-mutated ctDNA are represented in red and pink. The number in the box under “CA19-9” and “MctDNA” represents the CA19-9 value (U/mL) and the result of *KRAS*-mutated ctDNA analyses (copies/1 mL plasma). The negative threshold of copy number and CA19-9 were indicated as “< 5 copies” and “≦37”, respectively. Prognosis is shown under “Outcome”, with “Alive” and “Death” indicated in white and gray, respectively. Examination results for every three months are shown in one cell; thus, four cells correspond to approximately one year.

**Figure 2:**
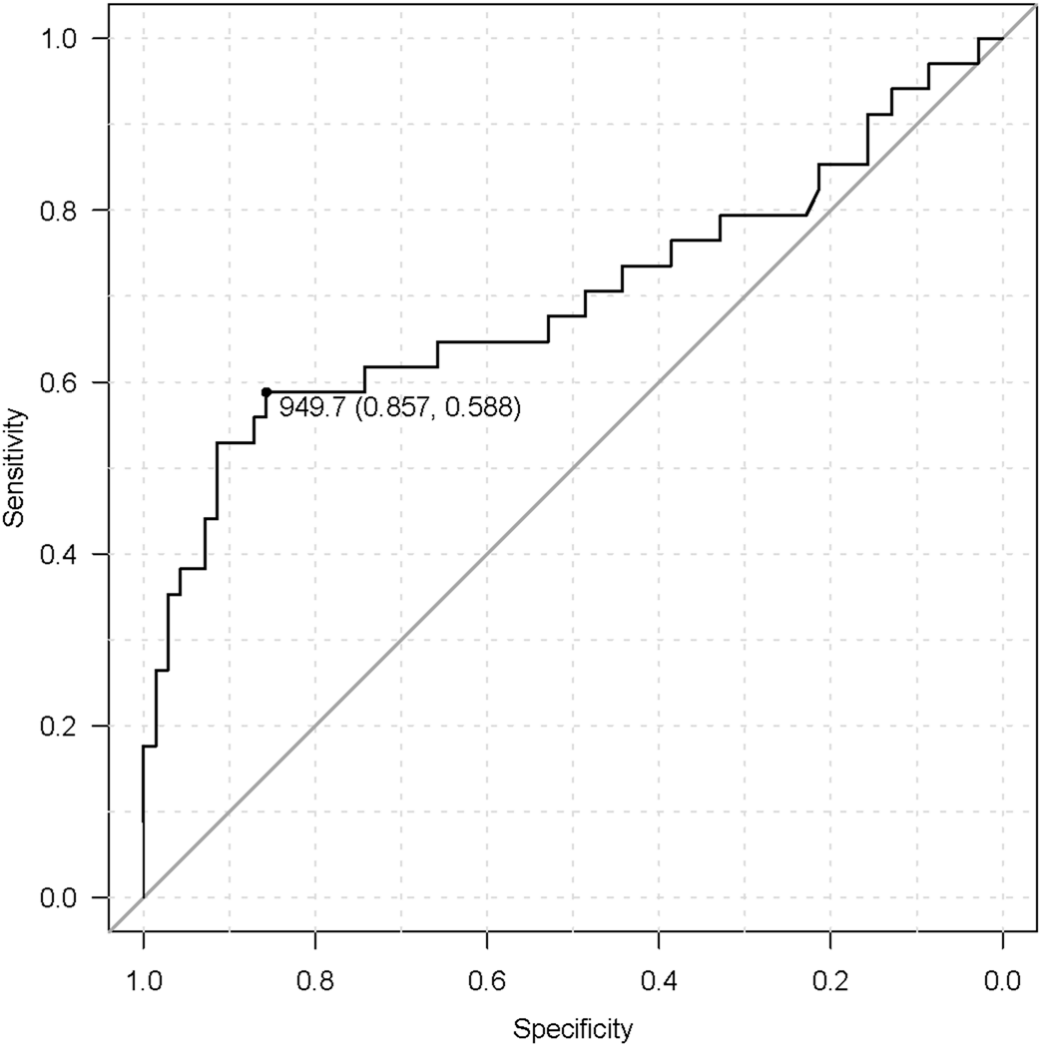
Receiver operating characteristics (ROC) analysis regarding the detection of *KRAS*-mutated ctDNA before chemotherapy in 22 patients considering all estimated points of ctDNA. ROC determined the cut-off value of CA19-9 at 949.7 U/mL to predict the presence of *KRAS*-mutated ctDNA in the blood during chemotherapy with a sensitivity and specificity of 58.8% and 85.7%, respectively. CA19-9 value ≥ 949.7 U/mL predicts the presence of *KRAS*-mutated ctDNA in the blood during chemotherapy with 58.8% sensitivity and 85.7% specificity.

Point mutations of *the KRAS* gene in tumor tissues were determined in advance using RASKET and ddPCR. Detected point mutations of *the KRAS* gene in tumor tissues were monitored in the blood of each patient who underwent chemotherapy. Therefore, no additional exploration of *KRAS* point mutations in ctDNA was required. Figure 1 shows the sequential assessments of *KRAS*-mutated ctDNA and CA19-9 in longitudinal monitoring. The ctDNA and CA19-9 levels were measured every three months after chemotherapy. During drug treatments, *KRAS*-mutated ctDNA was observed in 16 patients but not in the remaining six patients, while the elevation of CA19-9 level above the normal value was observed in 20 patients. Thirteen patients (59.1%) died due to disease progression. The emergence of *KRAS*-ctDNA in longitudinal monitoring was associated with a poor prognosis (*P* = 0.013). However, increased levels of CA19-9 were not associated with prognosis (*P* = 0.784). Details regarding the clinical course of the 22 patients who underwent chemotherapy are shown in Supplementary Table S1. Receiver operating characteristic (ROC) analysis was performed to determine the cutoff value of CA19-9 before chemotherapy in the 22 patients, corresponding to the detection of *KRAS*-mutated ctDNA (Supplementary Figure S1), which accounted for 1213.7 U/mL of CA19-9. Subsequently, we considered all estimated 104 samples of ctDNA from the 22 patients (22 samples before drug treatments and 82 samples during drug treatments) for ROC analysis, which identified 949.7 U/mL of CA19-9 as the cutoff value (Fig. 2). The sensitivity and specificity were 0.588 and 0.857, respectively. Patients who showed high levels of total bilirubin (≥ 3 mg/dL) were excluded from CA19-9 estimation. Supplementary Figure S2 shows the distribution of *KRAS*-mutated ctDNA detection in order of increasing levels of CA19-9 in all blood samples collected from the 22 patients who underwent chemotherapy. The detection rates of *KRAS*-mutated ctDNA in the blood were 65.5% (19/29) and 18.7% (14/75) in patients with CA19-9 > 1000 U/mL and those with CA19-9 ≤ 1000 U/mL, respectively (Supplementary Figure S2).

### Characteristics of patients who underwent surgery

In total, 104 patients underwent pancreatectomy for PDAC between 2005 and 2013. Forty-one patients (39.4%) were jaundiced or Lewis antigen-negative, and prognostic information was unavailable for three patients (2.9%); thus, these 44 patients were excluded from the evaluation. The remaining 60 patients were recruited for further analysis in this study; their overall characteristics are summarized in Table 2. The median CA19-9 level before surgery was 221.1 U/mL. Thirteen patients (21.7%) exhibited elevation of CA19-9 level to 949.7 U/mL or more before surgery.

**Table 2:** Characteristics of patients who underwent surgery.

Characteristics	Value (N = 60)
**Sex**	
Male	38 (63.3%)
Female	22 (36.7%)
**Age (median, 66.5 years)**	
≤ 67 years	30 (50.0%)
> 67 years	30 (50.0%)
**Tumor location**	
Head	41 (68.3%)
Body + tail	19 (31.7%)
**Tumor size**	
≤ 2 cm	11 (18.3%)
> 2 cm	49 (81.7%)
**UICC T**	
T1 + 2	36 (60.0%)
T3	24 (40.0%)
**Lymph node metastasis**	
Negative	18 (30.0%)
Positive	42 (70.0%)
**Pathological differentiation**	
G1 + G2	54 (90.0%)
Others	6 (10.0%)
**Resection margin**	
Negative	49 (82.0%)
Positive	9 (15.0%)
NA	2 (3.0%)
**CA19-9 (median, 221.05)**	
≤ 221.05 U/mL	30 (50.0%)
> 221.05 U/mL	30 (50.0%)
**CA19-9 (new cutoff level, 949.7)**	
≤ 949.7 U/mL	47 (78.3%)
> 949.7 U/mL	13 (21.7%)
**Adjuvant chemotherapy**	
No	16 (26.7%)
Yes	44 (73.3%)

Data are presented as number (percentage). UICC, Union for International Cancer Control; CA19-9, carbohydrate antigen 19-9; NA, not available.

### Outcome of patients who underwent surgery according to cutoff values of CA19-9

**Figure 3:**
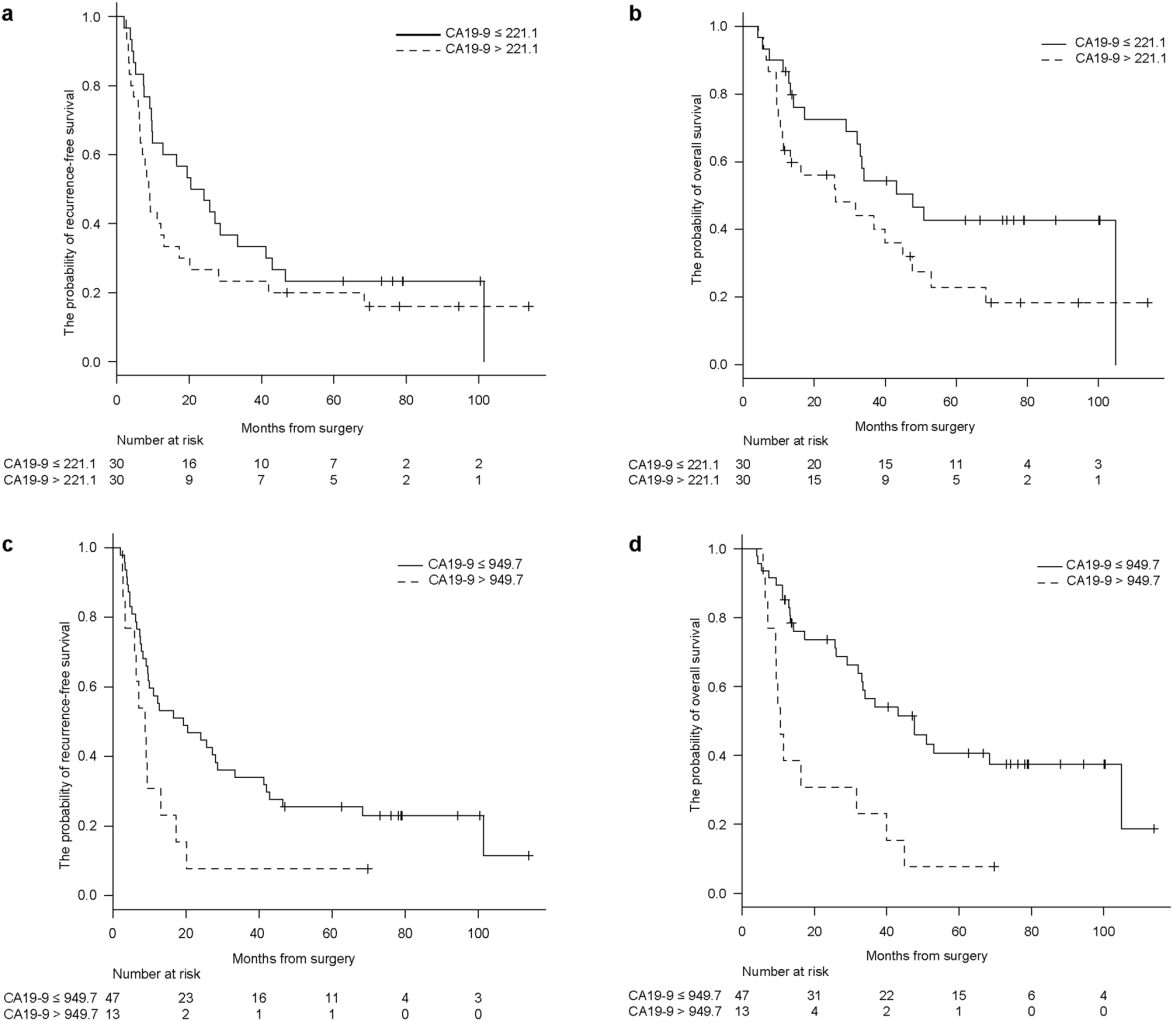
(**A, B**) Recurrence-free survival (RFS) and overall survival (OS) curves in patients who underwent surgery according to CA19-9 values (CA19-9 value ≥ 221.1 U/mL vs. CA19-9 value < 221.1 U/mL). The p-values were 0.21 and 0.15. The X-axis indicates the months from surgery, whereas the Y-axis indicates the probability of recurrence-free survival and overall survival. **(C, D)** Recurrence-free survival (RFS) and overall survival (OS) curves in patients who underwent surgery according to CA19-9 values (CA19-9 value ≥ 949.7 U/mL vs. CA19-9 value < 949.7 U/mL). The p-values were 0.020 and 0.001. The X-axis indicates the months from surgery, whereas the Y-axis indicates the probability of recurrence-free survival and overall survival.

Compared to the median value of CA19-9 (221.1 U/mL), the modified cutoff value of CA19-9 (949.7 U/mL) was assessed for its prognostic value of recurrence-free survival (RFS) and overall survival (OS) in 60 patients with resectable PDAC who underwent surgery. No significant difference in RFS and OS was observed between patients classified based on the median value of CA19-9 (*P* = 0.207 and *P* = 0.082, respectively; Fig. 3A, B). However, 949.7 U/mL of CA19-9 was linked to RFS and OS (*P* = 0.020 and *P* = 0.001, respectively; Fig. 3C, D). Interestingly, 949.7 U/mL of CA19-9 was also a predictor of recurrence and prognosis in 91 patients who underwent surgery, including 31 patients with jaundice (*P* = 0.030 and *P* = 0.002, respectively; Supplementary Figure S3A and B). Table 3 presents the 10 independent demographic and clinicopathological variables used in the univariate analysis for RFS of patients who underwent surgery. In addition to the value of 949.7 U/mL of CA19-9, lymph node metastasis, tumor size, and adjuvant chemotherapy were identified as potential recurrence factors (*P* = 0.020, *P* = 0.001, *P* = 0.030, and *P* = 0.066, respectively). Furthermore, five variables, including the value of 949.7 U/mL of CA19-9, lymph node metastasis, median CA19-9 value, tumor location, and tumor size, were identified as potential prognostic factors (*P* = 0.001, *P* = 0.0002, *P* = 0.0812, *P* = 0.020, and *P* = 0.004, respectively; Table 4). Multivariate Cox proportional hazards regression model indicated that the value of 949.7 U/mL of CA19-9 and lymph node metastasis were significant independent factors for recurrence and prognosis in 60 patients who underwent surgery (*P* = 0.008, *P* = 0.017, and *P* = 0.001, *P* = 0.010, respectively; Tables 3 and 4). In contrast, the median value of CA19-9 (221.1 U/mL) was not a predictor of recurrence or prognosis. An additional 31 patients with jaundice were included in the multivariate analysis. Multivariate analysis of 91 patients who underwent surgery showed that the value of 949.7 U/mL of CA19-9 was a predictor of prognosis rather than recurrence (*P* = 0.037 and *P* = 0.105, respectively; Supplementary Table S2A and B).

**Table 3:** Univariate and multivariate analyses of recurrence-free survival in the surgery group.

Prognostic factors	Univariate analysis			Multivariate analysis	
	No. of patients	MST (months)	*P*-value	Hazard ratio (95% CI)	*P*-value
**Sex**					
Male	38	10.385			
Female	22	18.9	0.362		
**Age (median, 66.5 years)**					
≤ 67 years	30	12.5			
> 67 years	30	11.385	0.357		
**Tumor location**					
Head	41	9.37			
Body + tail	19	28.1	0.182		
**Tumor size**					
≤ 2 cm	11	46.53		1	Reference
> 2 cm	49	9.930	0.0303	1.269 (0.4935–3.261)	0.6215
**T factor (UICC)**					
T1 + T2	36	19.815			
T3	24	8.73	0.124		
**Lymph node metastasis**					
Negative	18	43.9		1	Reference
Positive	42	9.05	0.00147	2.679 (1.1940–6.013)	0.016870
**Pathological differentiation**					
G1 + G2	54	12.5			
Others	6	15.83	0.621		
**Resection margin**					
Negative	49	16.6	0.205		
Positive	9	8.9			
**CA19-9 level**					
≤ 221.05 U/mL	30	22.265			
> 221.05 U/mL	30	9.015	0.207		
**CA19-9 level**					
≤ 949.7 U/mL	47	19.4		1	Reference
> 949.7 U/mL	13	8.9	0.0204	2.577 (1.2790–5.193)	0.008063
**Adjuvant chemotherapy**					
No	16	24.3		1	Reference
Yes	44	10.565	0.0659	1.385 (0.6717–2.855)	0.3778

MST, median survival time; CI, confidence interval; UICC, Union for International Cancer Control; CA19-9, carbohydrate antigen 19-9.

**Table 4:** Univariate and multivariate analyses of overall survival in surgery group.

Prognostic factors	Univariate analysis			Multivariate analysis	
	No. of patients	MST (months)	*P*-value	Hazard ratio (95% CI)	*P*-value
**Sex**					
Male	38	36.7			
Female	22	33.0	0.783		
**Age (median, 66.5 years)**					
≤ 67 years	30	33.0			
> 67 years	30	36.8	0.378		
**Tumor location**					
Head	41	26.0		1	Reference
Body + tail	19	68.4	0.02	0.4947 (0.2190–1.118)	0.090550
**Tumor size**					
≤ 2 cm	11	104.9		1	Reference
> 2 cm	49	31.7	0.00358	2.4260 (0.5970–9.856)	0.215400
**T factor (UICC)**					
T1 + T2	36	44.9			
T3	24	25.7	0.146		
**Lymph node metastasis**					
Negative	18	104.9		1	Reference
Positive	42	26.03	0.000277	3.9750 (1.3990–11.300)	0.009608
**Pathological differentiation**					
G1 + G2	54	36.77			
Others	6	21.93	0.555		
**Resection margin**					
Negative	49	34.0	0.181		
Positive	9	33.03			
**CA19-9 level**					
≤ 221.05 U/mL	30	47.7		1	Reference
> 221.05 U/mL	30	26.03	0.0815	0.5307 (0.2216–1.271)	0.155100
**CA19-9 level**					
≤ 949.7 U/mL	47	47.67		1	Reference
> 949.7 U/mL	13	10.53	0.000808	4.5650 (1.8180–11.470)	0.001228
**Adjuvant chemotherapy**					
No	16	47.67			
Yes	44	33	0.213		

MST, median survival rate; CI, confidence interval; UICC, Union for International Cancer Control; CA19-9, carbohydrate antigen 19-9; ctDNA, circulating tumor DNA.

## Discussion

This retrospective study proposed the optimal cutoff value of CA19-9 for predicting recurrence and prognosis in patients with resectable PDAC before surgery, which was attempted based on evidence from our previous study showing a correlation between the CA19-9 level and the presence of *KRAS*-mutated ctDNA in the blood of patients with unresectable PDAC^31^. The value of CA19-9 before surgery (949.7 U/mL) was an independent factor for the prediction of recurrence and prognosis in patients with resectable PDAC, while the median CA19-9 level was not predictive of recurrence or prognosis. To the best of our knowledge, no studies have determined the cutoff value of CA19-9 before surgery regarding the presence of *KRAS*-mutated ctDNA in the blood of patients with PDAC.

Increased levels of CA19-9 before surgery were reported to be associated with poorer prognosis after curative resection of PDAC in several single-institution retrospective studies^32–34^. Afterward, Hartwig et al. reported a correlation between serum levels of CA19-9 and tumor stages in a large cohort of more than 1600 patients with PDAC, including early localized to metastatic disease, and identified progressively decreasing 5-year survival rates and median survival times with increasing CA19-9 levels, up to a CA19-9 level of 1000 U/mL^35^. Montgomery et al. found a longer median survival time of 34 months versus 16 months for patients with a preoperative value of less than 1052 U/mL (*P* < 0.018)^18^. Therefore, we expected that the optimal cutoff value of CA19-9 for predicting prognosis would be approximately 1,000 U/mL and demonstrated that 949.7 U/mL of CA19-9 was significantly associated with the presence of *KRAS*-mutated ctDNA. Nakao et al. reported that 13 of 15 patients with a preoperative CA19-9 value > 2000 U/mL survived less than 24 months after resection^19^. Our current study showed an increase in median survival time of 47.7 months in patients with preoperative CA19-9 values less than 949.7 U/mL compared to that of 10.5 months in patients with preoperative CA19-9 values of 949.7 U/mL or more (*P* = 0.001). These data suggest that patients with an extremely high level of CA19-9 before surgery are more likely to have a larger tumor burden, which cannot be easily detected by routine imaging studies, resulting in a poor prognosis. Therefore, indications for complex and complication-bearing several vessels pancreatectomies should be considered with caution in patients with extremely high CA19-9 levels because the benefit-to-risk ratio may be marginal. A recent study proposed that CA19-9 alterations during neoadjuvant chemotherapy may help select patients who will benefit from radical resection^36^. Gemcitabine + TS1 therapy is widely used as neoadjuvant chemotherapy for resectable pancreatic cancer in Japan. However, powerful anticancer drug regimens, such as FOLFIRINOX, could be an alternative for gemcitabine + TS1 therapy depending on the level of elevation in CA19-9.

The values of CA19-9 were disregarded in PDAC patients with hyperbilirubinemia, while more than half of patients with pancreatic head cancers presented with jaundice. Altered biliary excretion, for which bilirubin is a useful marker, has been documented to occur at levels 1.5 × the upper limit of normal or at a level of approximately 2.0 mg/dL^37^. In our study, patients with serum bilirubin levels > 3 mg/dL were excluded from the estimation of CA19-9 before surgery. Kang et al. showed that preoperative levels of CA19-9, adjusted by the level of serum bilirubin, were predictive of survival after resection of pancreatic cancer; however, the study did not assess whether the adjusted value of CA19-9 was more or less predictive than the unadjusted value^38^. Our analysis showed that 949.7 U/mL of CA19-9 before surgery could also be used to predict prognosis even in PDAC patients with hyperbilirubinemia (Supplementary Figure S3A and B). Many histopathological factors were investigated in relation to the prognostic outcomes of patients with PDAC, including tumor size, number of lymph nodes, tumor grade, stage, and margin status of the resected specimen. Lymph node metastasis was considered an important factor for predicting OS and RFS in patients with non-metastatic pancreatic cancer who underwent surgery^39,40^, which is consistent with our study. At present, most patients with PDAC are estimated for staging using contrast-enhanced spiral computed tomography (CT). CT imaging has a high specificity for predicting unresectability; however, its sensitivity is poor^5,41–45^, potentially resulting in many patients undergoing unnecessary surgery, similar to magnetic resonance imaging (MRI)^46,47^. Many studies have reported that ctDNA reflects tumor dynamics in many carcinomas, which is consistent with our previous studies monitoring *KRAS*-mutated ctDNA in pancreatic and colorectal cancer^30,31^. The importance of longitudinal monitoring has been addressed for predicting the outcome of PDAC to detect the emergence of ctDNA^48,49^ along with dynamic changes in tumor markers in various cancers, including CA19-9. Tjensvoll et al. reported that changes in the levels of *KRAS* ctDNA in the circulation correlated with the levels of CA19-9 during chemotherapy^50^, suggesting that extraordinarily high CA19-9 levels may represent micrometastasis, including lymph node metastasis, which is difficult to detect via imaging studies before surgery. With accurate preoperative staging before surgery, unnecessary surgical treatment should be avoided.

The prognostic difference between *mtKRAS* and *wtKRAS* PDAC should be addressed. In the chemotherapy cohort, the detection of *KRAS* tumor mutations was performed using the RASKET method and ddPCR in 22 patients. In addition, the RASKET method revealed only one patient harboring the wild-type gene (Case no. 6 in Supplementary Table S1). Due to the small proportion of patients with *wtKRAS* PDAC, we did not demonstrate the contribution of *KRAS* status to the prognosis or treatment outcome in the chemotherapy cohort. Eventually, a 12D mutation was identified in this patient by ddPCR.

Some limitations associated with the present study warrant mention. First, this was a retrospective cohort study conducted at a single institution, and the number of enrolled patients was relatively small. Second, because of the long duration of patient enrollment, the policy and regimens concerning adjuvant chemotherapy and neoadjuvant chemotherapy changed.

In summary, our study demonstrated, for the first time, that the adjusted cutoff value of CA19-9 was an important biomarker for the prediction of recurrence and prognosis in patients with resected PDAC. Patients with high levels of CA19-9 before surgery had a higher risk of recurrence and poorer prognosis than those with low levels of CA 19-9; they may have benefitted more from drug treatment than surgical intervention. Nevertheless, appropriate clinical decision-making for patients with high CA 19-9 levels should be confirmed in future clinical trials. Although our study results should be interpreted within the study limitations and further examinations are required to draw a definitive conclusion, we believe that it clarifies the selection of patients with PDAC.

## Methods

### Patients and study design

We prospectively collected data from 22 patients who were diagnosed with unresectable PDAC. They underwent chemotherapy between June 2015 and December 2017 at Saitama Medical Center, Jichi Medical University, Japan. One hundred and four blood samples collected from the 22 patients were available for assessments, including 22 and 82 samples before and during drug treatments, respectively. In addition, we retrospectively collected data from 104 patients with PDAC who underwent surgery between March 2005 and March 2013 at our hospital. The characteristics of the 22 and 104 patients who underwent chemotherapy and 104 patients who underwent surgery are shown in Supplementary Tables S1 and S3, respectively. Of the 104 patients with PDAC who underwent surgery, three were not diagnosed with PDAC, 10 were suspected of being Lewis antigen-negative, and 31 patients with hyperbilirubinemia (bilirubin level of 3 mg/dL or more) were excluded from the evaluation of CA19-9 regardless of the presence or absence of bile drainage. Consequently, the remaining 60 patients were enrolled in the evaluation. In this study, patients with 2 U/mL of CA19-9 or less were defined as Lewis antibody-positive. After surgery, evidence of recurrence was confirmed based on imaging findings. The median follow-up time for patients in the surgery group was 22.7 months. All patients provided written informed consent to examine their tissue and plasma and the use of their clinical data. The study protocol was approved by the research ethics committee of Jichi Medical University and conformed to the ethical guidelines of the World Medical Association Declaration of Helsinki.

### Analysis of *KRAS* status in PDAC tissues

*KRAS* status in PDAC tissues of the chemotherapy group was evaluated by RASKET with a sensitivity of 1–5%^51^ and ddPCR with a sensitivity of 0.01–0.1%^52,53^, using endoscopic ultrasound-guided fine-needle aspiration samples. *KRAS* status was analyzed in 22 tumor tissues by a clinical testing company (Special Reference Laboratories, Tokyo, Japan) using RASKET. Tissue DNA was extracted from 22 formalin-fixed paraffin-embedded (FFPE) tissues using the QIAamp DNA FFPE Tissue Kit (Qiagen, Hilden, Germany) according to the manufacturer’s instructions. Early reports showed that point mutations at codon 12 of the *KRAS* oncogene mostly include G12V, G12D, and G12R, while other types of *KRAS* point mutations are rarely detected in patients with PDAC^54–56^. Therefore, these three types of *KRAS* mutations were predominantly identified by ddPCR. In addition, Q61H, another type of *KRAS* mutation that emerged prior to drug resistance, was verified in four patients by ddPCR after initial determination by RASKET. *KRAS* status in two patients could not be assessed because of insufficient DNA samples.

### Plasma sample collection and processing

In total, 106 blood samples were collected from patients with locally advanced and metastatic PDAC in the chemotherapy group at Saitama Medical Center, Jichi Medical University. From each patient, 7 mL of whole blood was drawn into EDTA-containing tubes, and plasma was collected by centrifugation at 3000×*g* for 20 min at 4 °C, followed by centrifugation at 16,000×*g* for 10 min at 4 °C in a fresh tube. Plasma samples were separated from peripheral blood cells and stored at -80 °C until DNA extraction.

### Extraction of circulating cell-free DNA

Circulating cell-free DNA was extracted from 2 mL of plasma using the QIAamp Circulating Nucleic Acid Kit (Qiagen, Hilden, Germany) according to the manufacturer’s instructions and eluted in 80 µL of elution, in which 10 µL of elution was administrated in the 20 µL of ddPCR reaction.

### Droplet digital polymerase chain reaction analyses

*KRAS* status in tumor tissues and plasma was analyzed using the Bio-Rad QX200 ddPCR system (Bio-Rad Laboratories, Hercules, CA, USA). We used a commercially available PrimePCR *KRAS* kit for ddPCR (Bio-Rad Laboratories, Hercules, CA, USA). *KRAS* mutations in each blood sample were verified according to the corresponding mutation (C12V, G12D, G12R, and Q61H) in matched tumor tissues determined by ddPCR. The reaction mixture comprised 10 µL of 2 × ddPCR Supermix, 1 µL of each reference and variant 20 × Bio-Rad PrimePCR *KRAS* for ddPCR, and 10 µL of sample eluted from plasma in a final volume of 22 µL. The mixture was loaded onto a DG8 cartridge (Bio-Rad Laboratories, Hercules, CA, USA) with 70 µL of droplet generation oil, and the cartridge was placed into a droplet generator. The generated droplets (approximately 15,000 generated droplets per well) were transferred to a 96-well reaction plate, heat-sealed with a foil lid, and subjected to thermocycling in a Veriti thermal cycler (Thermo Fisher Scientific, Waltham, MA, USA) under the following cycling conditions: 95 °C for 10 min and 40 cycles at 95 °C for 30 s and 55 °C for 90 s. Amplified droplets were analyzed using a QX200 droplet reader (Bio-Rad Laboratories, Hercules, CA, USA) for the fluorescence measurement of FAM and HEX probes for wild-type and mutant genes, respectively. QuantaSoft software (Bio-Rad Laboratories, Hercules, CA, USA) was used to measure the number of positive and negative droplets. Samples with two or more positive droplets were considered positive according to the threshold values, as previously reported^30^. For data reproducibility, analysis of *KRAS* status in tumor tissues and plasma was performed in duplicate or triplicate.

Furthermore, ddPCR is comprised of approximately 20,000 partitioned droplets. The sample is randomly distributed into discrete partitioned droplets, such that some contain no nucleic acid template and others contain one or more template copies. The partitioned droplets are thermally cycled to the endpoint and then read to determine the fraction of positive droplets, from which the concentration is calculated using the following formula^57^.

$$ {\text{M}} = \, - {\text{ln}}\left( {{1} - \left( {{\text{P}}/{\text{R}}} \right)} \right){\text{ copy number per droplet}} $$

M is the average number of target molecules per droplet, in other words, the average copy number per droplet. P is the number of droplets containing amplified product, and R is the number of droplets or reactions analyzed. R varies in each reaction of samples (for example, 20,000 droplets and 15,000 droplets). If 20,000 droplets were analyzed, the average copy number in one positive droplet per well is calculated as follows,

$$ {\text{M }} = - {\text{ln}}\left( {{1} - \left( {{\text{P}}/{\text{R}}} \right)} \right) \, = - {\text{ln}}\left( {{1} - \left( {{1}/{2}0000} \right)} \right) \, = 0.0000{\text{5 copies}}/{\text{droplet}}, $$

which contains 0.05 copies/µL in the 20 µL ddPCR reaction in one well. One droplet contains 1 copy in one reaction.

Circulating cell-free DNA was extracted from 2 mL of plasma and eluted in 80 µL of elution, in which 10 µL of elution was administrated in the 20 µL of ddPCR reaction. One droplet contains 4 copies in 1 mL of plasma. Therefore, < 5 copies/ml plasma is set as the threshold value of negative droplets instead of the number of droplets.

### Statistical analysis

ROC curve analysis was plotted to determine the cutoff value of CA19-9 corresponding to the presence of *KRAS*-mutated ctDNA. To assess prognosis in the surgery group, we measured RFS and OS as endpoints. RFS was defined as the time from surgery to confirmation of recurrence based on radiological findings. OS was defined as the time from surgery to event occurrence. A Cox proportional hazards regression model was used to evaluate the association between overall mortality and other factors in univariate and multivariate analyses. The following variables were analyzed in patients: sex; age at surgery (≤ 67 years vs. > 67 years); adjuvant chemotherapy (yes versus no); tumor location (head versus body and tail); tumor size (≤ 2 cm vs.  > 2 cm); pathological differentiation (well and moderate versus others); Union for International Cancer Control (UICC) T factor (T1 + T2 vs. T3), lymph node metastasis (negative versus positive), preoperative CA19-9 level (≤ median CA19-9 value, 221.1 U/mL versus > median CA19-9 value, 221.1 U/mL); and preoperative CA19-9 level (≤ new cutoff of CA19-9 value; 949.7 U/mL versus > new cutoff of CA19-9 value; 949.7 U/mL). RFS and OS curves were constructed using the Kaplan–Meier method. Several factors with a *P-*value of < 0.1 in univariate analysis were subjected to multivariate analysis. Statistical significance was set at *P* < 0.05. Fisher’s exact test was used for categorical variables, such as the presence of *KRAS-*mutated ctDNA, CA19-9 level (≥ 37 U/mL vs. < 37 U/mL), and outcome (dead or alive). All statistical analyses were performed using EZR version 1.31 (Saitama Medical Center, Jichi Medical University, Saitama, Japan). R version 3.1.1 (The R Foundation for Statistical Computing, Vienna, Austria) was used for the graphical interface.

## Supplementary Information

Supplementary Information 1.

Supplementary Figure S1.

Supplementary Figure S2.

Supplementary Figure S3.

Supplementary Table S1.

Supplementary Table S2.

Supplementary Table S3.

## Data Availability

All data generated or analyzed during this study are included in this published article (and its Supplementary Information files).

## Abbreviations

AJCC: American Joint Committee on Cancer
CA19-9: Carbohydrate antigen 19-9
ctDNA: Circulating tumor DNA
ddPCR: Droplet digital polymerase chain reaction
FFPE: Formalin-fixed paraffin-embedded
*KRAS*: Kirsten rat sarcoma viral oncogene homolog
OS: Overall survival
PDAC: Pancreatic ductal adenocarcinoma
PFS: Progression-free survival
RECIST: Response evaluation criteria in solid tumors
RFS: Recurrence-free survival

## References

[1] Bray F (2018). Global cancer statistics 2018: GLOBOCAN estimates of incidence and mortality worldwide for 36 cancers in 185 countries. CA Cancer J. Clin..

[2] Rahib L (2014). Projecting cancer incidence and deaths to 2030: The unexpected burden of thyroid, liver, and pancreas cancers in the United States. Cancer Res..

[3] Strobel O, Neoptolemos J, Jager D, Buchler MW (2019). Optimizing the outcomes of pancreatic cancer surgery. Nat. Rev. Clin. Oncol..

[4] Singh P, Srinivasan R, Wig JD (2011). Major molecular markers in pancreatic ductal adenocarcinoma and their roles in screening, diagnosis, prognosis, and treatment. Pancreas.

[5] Barugola G (2009). Resectable pancreatic cancer: Who really benefits from resection?. Ann. Surg. Oncol..

[6] Hess V (2008). CA 19-9 tumour-marker response to chemotherapy in patients with advanced pancreatic cancer enrolled in a randomised controlled trial. Lancet Oncol..

[7] Humphris, J. L. *et al.* The prognostic and predictive value of serum CA19.9 in pancreatic cancer. *Ann. Oncol.* **23**, 1713–1722. 10.1093/annonc/mdr561 (2012). 10.1093/annonc/mdr561 PMC3387824 22241899

[8] Karachristos A, Scarmeas N, Hoffman JP (2005). CA 19-9 levels predict results of staging laparoscopy in pancreatic cancer. J. Gastrointest. Surg..

[9] Maithel SK (2008). Preoperative CA 19-9 and the yield of staging laparoscopy in patients with radiographically resectable pancreatic adenocarcinoma. Ann. Surg. Oncol..

[10] Wasan HS (2009). CA 19-9 as a biomarker in advanced pancreatic cancer patients randomised to gemcitabine plus axitinib or gemcitabine alone. Br. J. Cancer.

[11] Marchegiani G (2017). Does size matter in pancreatic cancer?: Reappraisal of tumour dimension as a predictor of outcome beyond the TNM. Ann. Surg..

[12] Ferrone CR (2006). Perioperative CA19-9 levels can predict stage and survival in patients with resectable pancreatic adenocarcinoma. J. Clin. Oncol..

[13] Dong Q (2014). Elevated serum CA19-9 level is a promising predictor for poor prognosis in patients with resectable pancreatic ductal adenocarcinoma: A pilot study. World J. Surg. Oncol..

[14] Imaoka H (2016). Post-adjuvant chemotherapy CA19-9 levels predict prognosis in patients with pancreatic ductal adenocarcinoma: A retrospective cohort study. Pancreatology.

[15] Laurent L (2019). CA19.9 decrease >15% is a predictor of favourable outcome in patients treated for advanced pancreatic carcinoma: Analysis of two independent cohorts. HPB (Oxford).

[16] Robert M (2017). Retrospective analysis of CA19-9 decrease in patients with metastatic pancreatic carcinoma treated with FOLFIRINOX or gemcitabine in a randomized phase III study (ACCORD11/PRODIGE4). Oncology.

[17] Tsai S (2020). Importance of normalization of CA19-9 levels following neoadjuvant therapy in patients with localized pancreatic cancer. Ann. Surg..

[18] Montgomery RC (1997). Prediction of recurrence and survival by post-resection CA 19-9 values in patients with adenocarcinoma of the pancreas. Ann. Surg. Oncol..

[19] Nakao A (1998). Clinical usefulness of CA-19-9 in pancreatic carcinoma. Semin. Surg. Oncol..

[20] Ballehaninna UK, Chamberlain RS (2012). The clinical utility of serum CA 19-9 in the diagnosis, prognosis and management of pancreatic adenocarcinoma: An evidence based appraisal. J. Gastrointest. Oncol..

[21] Jones NB (2009). Clinical factors predictive of malignant and premalignant cystic neoplasms of the pancreas: A single institution experience. HPB (Oxford).

[22] Sang X (2011). Hepatobiliary cystadenomas and cystadenocarcinomas: A report of 33 cases. Liver Int..

[23] De Mattos-Arruda L, Olmos D, Tabernero J (2011). Prognostic and predictive roles for circulating biomarkers in gastrointestinal cancer. Future Oncol..

[24] Gormally E, Caboux E, Vineis P, Hainaut P (2007). Circulating free DNA in plasma or serum as biomarker of carcinogenesis: Practical aspects and biological significance. Mutat. Res..

[25] Iede, K. *et al.* Predictive implications of decreased CA19-9 at 8 weeks during nab-paclitaxel plus gemcitabine for the induction of second-line chemotherapy for patients with advanced pancreatic cancer. *Cancer Rep. (Hoboken)* **3**, e1289. 10.1002/cnr2.1289 (2020). 10.1002/cnr2.1289 PMC7941508 32969199

[26] Bettegowda, C. *et al.* Detection of circulating tumor DNA in early- and late-stage human malignancies. *Sci. Transl. Med.* **6**, 224ra224. 10.1126/scitranslmed.3007094 (2014). 10.1126/scitranslmed.3007094 PMC4017867 24553385

[27] Mead R, Duku M, Bhandari P, Cree IA (2011). Circulating tumour markers can define patients with normal colons, benign polyps, and cancers. Br. J. Cancer.

[28] Kamat AA (2010). Plasma cell-free DNA in ovarian cancer: An independent prognostic biomarker. Cancer.

[29] Diehl F (2008). Circulating mutant DNA to assess tumor dynamics. Nat. Med..

[30] Takayama Y (2018). Monitoring circulating tumor DNA revealed dynamic changes in KRAS status in patients with metastatic colorectal cancer. Oncotarget.

[31] Watanabe F (2019). Longitudinal monitoring of KRAS-mutated circulating tumor DNA enables the prediction of prognosis and therapeutic responses in patients with pancreatic cancer. PLoS ONE.

[32] Kondo N (2010). Prognostic impact of perioperative serum CA 19-9 levels in patients with resectable pancreatic cancer. Ann. Surg. Oncol..

[33] Barton JG (2009). Predictive and prognostic value of CA 19-9 in resected pancreatic adenocarcinoma. J. Gastrointest. Surg..

[34] Turrini O (2009). Very high serum CA 19-9 levels: A contraindication to pancreaticoduodenectomy?. J. Gastrointest. Surg..

[35] Hartwig W (2013). CA19-9 in potentially resectable pancreatic cancer: Perspective to adjust surgical and perioperative therapy. Ann. Surg. Oncol..

[36] Aoki S (2019). Decreased serum carbohydrate antigen 19-9 levels after neoadjuvant therapy predict a better prognosis for patients with pancreatic adenocarcinoma: A multicenter case-control study of 240 patients. BMC Cancer.

[37] Schlieman, M. G., Ho, H. S. & Bold, R. J. Utility of tumor markers in determining resectability of pancreatic cancer. *Arch. Surg.* **138**, 951–955; discussion 955–956. 10.1001/archsurg.138.9.951 (2003). 10.1001/archsurg.138.9.951 12963650

[38] Kang CM (2007). The use of adjusted preoperative CA 19-9 to predict the recurrence of resectable pancreatic cancer. J. Surg. Res..

[39] Brennan MF, Kattan MW, Klimstra D, Conlon K (2004). Prognostic nomogram for patients undergoing resection for adenocarcinoma of the pancreas. Ann. Surg..

[40] Karjol U (2020). Lymph node ratio as a prognostic marker in pancreatic cancer survival: A systematic review and meta-analysis. Cureus.

[41] Ramsay D (2004). Identification and staging of pancreatic tumours using computed tomography, endoscopic ultrasound and *Mangafodipir trisodium*-enhanced magnetic resonance imaging. Australas. Radiol..

[42] Bluemke DA (1995). Potentially resectable pancreatic adenocarcinoma: Spiral CT assessment with surgical and pathologic correlation. Radiology.

[43] Trede, M. *et al.* Ultrafast magnetic resonance imaging improves the staging of pancreatic tumors. *Ann Surg* **226**, 393–405; discussion 405–397. 10.1097/00000658-199710000-00001 (1997). 10.1097/00000658-199710000-00001 PMC1191049 9351708

[44] Nakayama Y (2001). Vascular encasement by pancreatic cancer: Correlation of CT findings with surgical and pathologic results. J. Comput. Assist. Tomogr..

[45] Lu DS, Reber HA, Krasny RM, Kadell BM, Sayre J (1997). Local staging of pancreatic cancer: criteria for unresectability of major vessels as revealed by pancreatic-phase, thin-section helical CT. AJR Am. J. Roentgenol..

[46] Lopez Hänninen, E. *et al.* Prospective evaluation of pancreatic tumors: accuracy of MR imaging with MR cholangiopancreatography and MR angiography. *Radiology* **224**, 34–41. 10.1148/radiol.2241010798 (2002). 10.1148/radiol.2241010798 12091659

[47] Megibow AJ (1995). Pancreatic adenocarcinoma: CT versus MR imaging in the evaluation of resectability–report of the Radiology Diagnostic Oncology Group. Radiology.

[48] Bernard, V. *et al.* Circulating nucleic acids are associated with outcomes of patients with pancreatic cancer. *Gastroenterology* **156**, 108–118 e104. 10.1053/j.gastro.2018.09.022 (2019). 10.1053/j.gastro.2018.09.022 PMC6434712 30240661

[49] Sausen M (2015). Clinical implications of genomic alterations in the tumour and circulation of pancreatic cancer patients. Nat Commun.

[50] Tjensvoll K (2016). Clinical relevance of circulating KRAS mutated DNA in plasma from patients with advanced pancreatic cancer. Mol Oncol.

[51] Yoshino T (2015). Clinical validation of a multiplex kit for RAS mutations in colorectal Cancer: Results of the RASKET (RAS KEy Testing) Prospective Multicenter Study. EBioMedicine.

[52] Hindson BJ (2011). High-throughput droplet digital PCR system for absolute quantitation of DNA copy number. Anal. Chem..

[53] Watanabe M (2015). Ultra-sensitive detection of the pretreatment EGFR T790M mutation in non-small cell lung cancer patients with an EGFR-activating mutation using droplet digital PCR. Clin. Cancer Res..

[54] Chen H (2010). K-ras mutational status predicts poor prognosis in unresectable pancreatic cancer. Eur. J. Surg. Oncol..

[55] Witkiewicz AK (2015). Whole-exome sequencing of pancreatic cancer defines genetic diversity and therapeutic targets. Nat. Commun..

[56] Yachida S (2012). Clinical significance of the genetic landscape of pancreatic cancer and implications for identification of potential long-term survivors. Clin. Cancer Res..

[57] Pinheiro LB (2012). Evaluation of a droplet digital polymerase chain reaction format for DNA copy number quantification. Anal. Chem..

