# Supplementary material for: Optimal value of CA19-9 determined by KRAS-mutated circulating tumor DNA contributes to the prediction of prognosis in pancreatic cancer patients
Source: Sci Rep. 2021 Oct 21;11:20797. doi: 10.1038/s41598-021-00060-9 (PMC8531317; doi:10.1038/s41598-021-00060-9)
Supplement: Supplementary file 1 — Supplementary Information 1. [file 41598_2021_60_MOESM1_ESM.docx]

Optimal value of CA19-9 determined by *KRAS*-mutated circulating tumor DNA contributes to the prediction of prognosis in pancreatic cancer patients

Fumiaki Watanabe, Koichi Suzuki, Sawako Tamaki, Iku Abe, Yuhei Endo, Yuji Takayama, Hideki Ishikawa, Nao Kakizawa, Masaaki Saito, Kazushige Futsuhara, Hiroshi Noda, Fumio Konishi, Toshiki Rikiyama

**Supporting information**

**Supplementary Figure S1. Receiver operating characteristics (ROC) curve regarding the detection of *KRAS*-mutated ctDNA before chemotherapy in 22 patients.** The sensitivity and specificity were 58.8% and 85.7%, respectively.

**Supplementary Figure S2. Distribution of the detection of *KRAS-*mutated ctDNA in relation to increasing levels of CA 19-9 in all blood samples collected from 22 patients who underwent chemotherapy.**

The x-axis indicates blood samples in order of increasing levels of CA 19-9. The y-axis indicates the level of CA19-9. The upper limit of CA19-9 level was set to 1000 U/mL. The black box shows the samples wherein *KRAS*-mutated ctDNA was detected, and the white box shows the samples wherein no *KRAS*-mutated ctDNA was detected.

**Supplementary Figure S3A. Recurrence-free survival curve in patients who underwent surgery including those with jaundice according to the CA19-9 value (CA19-9 value ≥ 949.7 U/mL vs. CA19-9 value <949.7 U/mL).**

The p-value is 0.0302. The x-axis indicates months post-surgery. The y-axis indicates the probability of recurrence-free survival.

**Supplementary Figure S3B. Overall survival curve in patients who underwent surgery including those with jaundice according to the CA19-9 value (CA19-9 value ≥ 949.7 U/mL vs. CA19-9 value <949.7 U/mL).**

The p-value is 0.00201. The x-axis indicates months post-surgery. The y-axis indicates the probability of overall survival.
