# Supplementary figures and images for: Optimal value of CA19-9 determined by KRAS-mutated circulating tumor DNA contributes to the prediction of prognosis in pancreatic cancer patients
Source: Sci Rep. 2021 Oct 21;11:20797. doi: 10.1038/s41598-021-00060-9 (PMC8531317; doi:10.1038/s41598-021-00060-9)

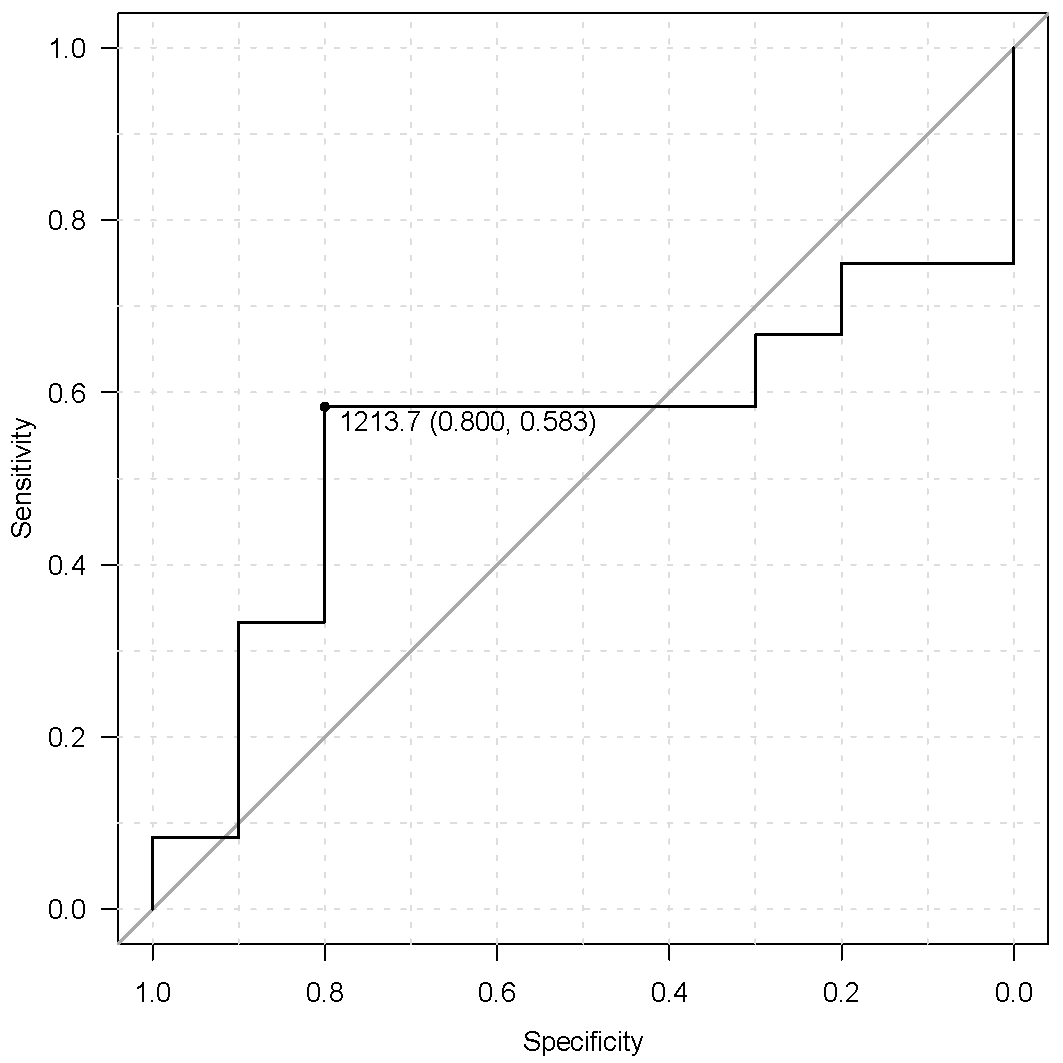

Supplement: Supplementary file 2 — Supplementary Figure S1. [file 41598_2021_60_MOESM2_ESM.tif]

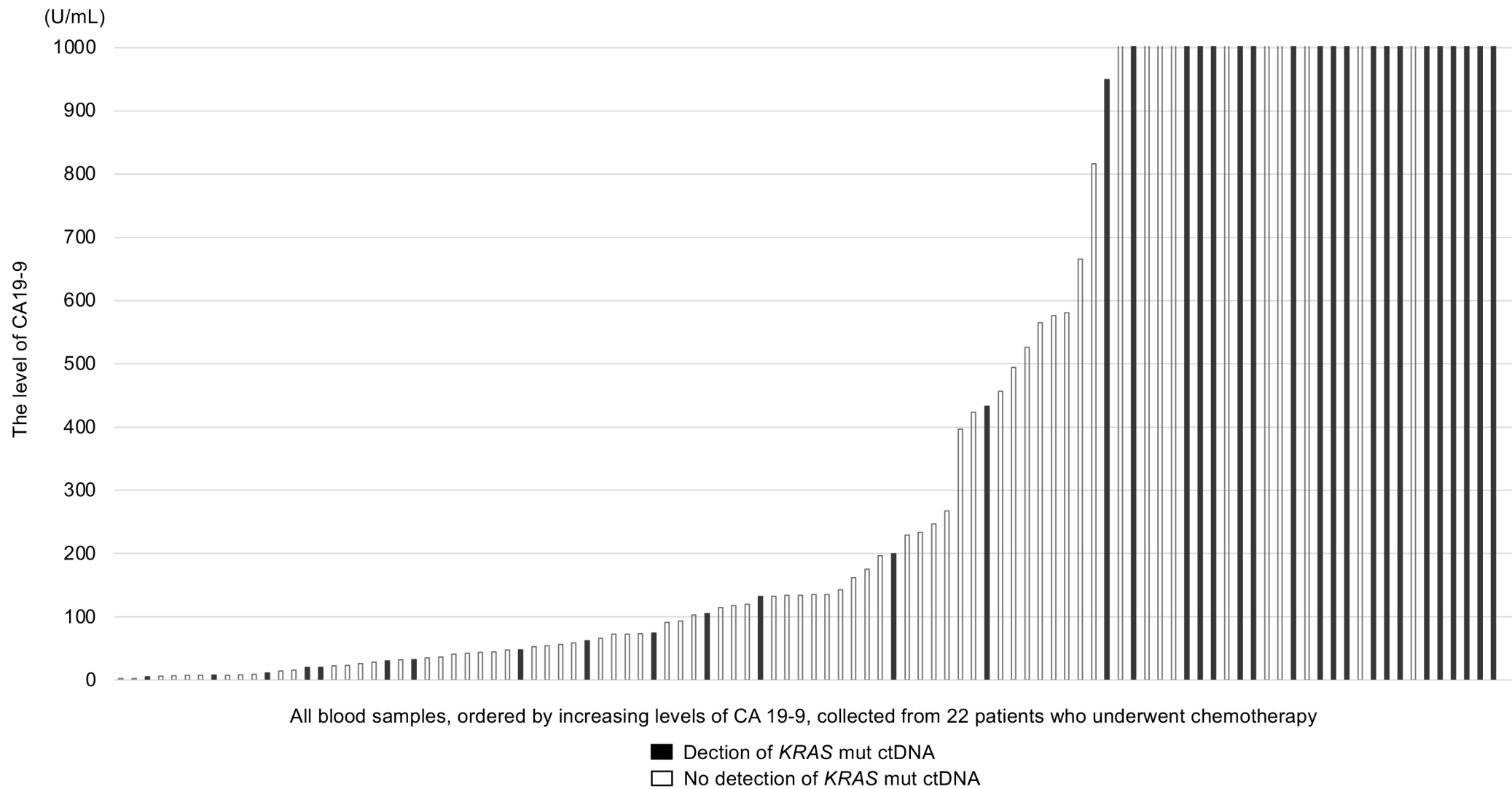

Supplement: Supplementary file 3 — Supplementary Figure S2. [file 41598_2021_60_MOESM3_ESM.pdf]

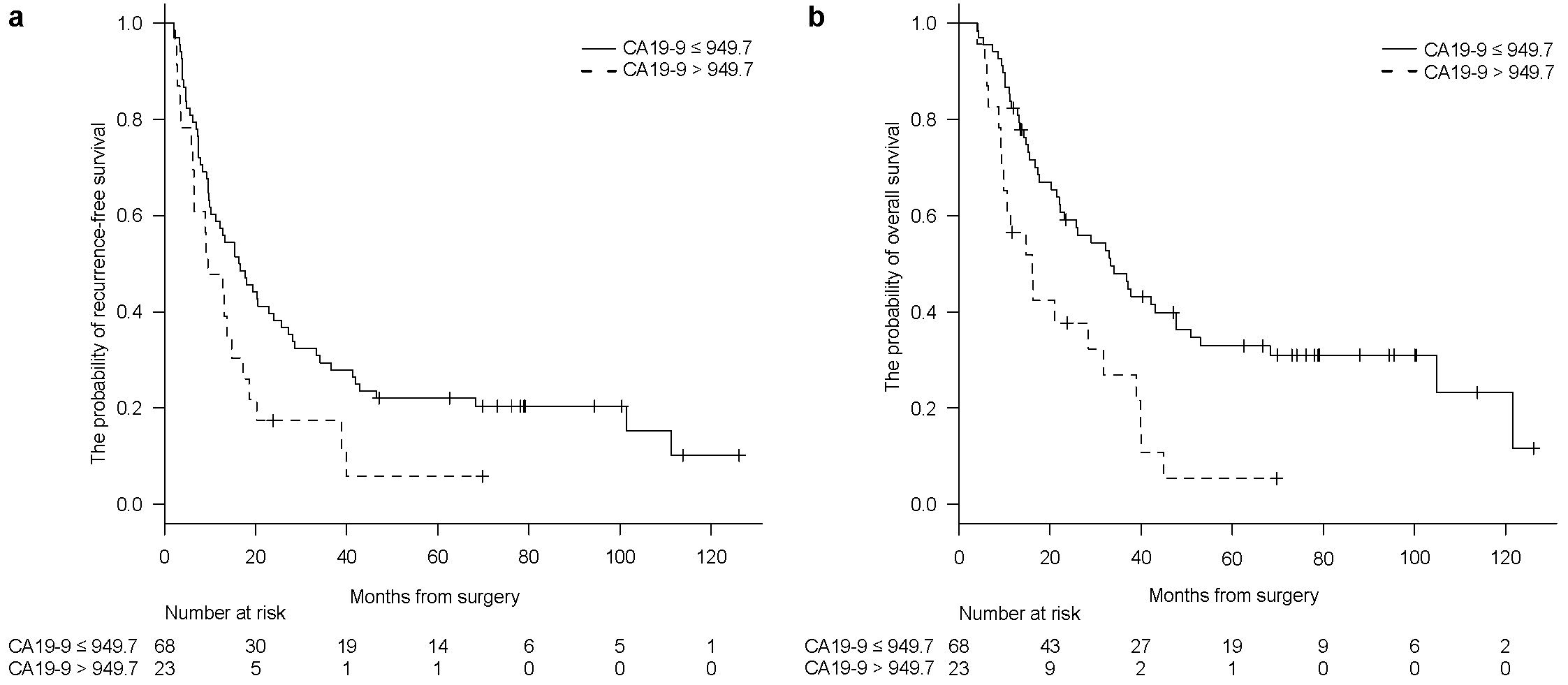

Supplement: Supplementary file 4 — Supplementary Figure S3. [file 41598_2021_60_MOESM4_ESM.tif]
