# Supplementary material for: Optimal value of CA19-9 determined by KRAS-mutated circulating tumor DNA contributes to the prediction of prognosis in pancreatic cancer patients
Source: Sci Rep. 2021 Oct 21;11:20797. doi: 10.1038/s41598-021-00060-9 (PMC8531317; doi:10.1038/s41598-021-00060-9)
Supplement: Supplementary file 5 — Supplementary Table S1. [file 41598_2021_60_MOESM5_ESM.docx]

**Supplementary Table S1.** Clinical information of patients who underwent chemotherapy

| Patients | Sex | Age | Unresectable factor | *KRAS* mutation | | CA19-9 level before chemotherapy | Emergence of *KRAS*-mutated ctDNA before chemotherapy | Emergence of *KRAS-* mutated ctDNA | Chemotherapy |
| --- | --- | --- | --- | --- | --- | --- | --- | --- | --- |
|  |  | (years) |  | In tissue by RASKET | In tissue by ddPCR |  |  | In monitoring | First-line |
| 1 | m | 56 | Locally advanced | 12V | 12V | 456.8 |  |  | FOLFIRINOX |
| 2 | f | 65 | Locally advanced | ND | 12V, 12D | 132 | 12V | 12V | GnP |
| 3 | f | 65 | LN metastasis | 12V | 12V, 12D | 11.1 | 12V | 12V | FOLFIRINOX |
| 4 | m | 76 | Locally advanced, lung metastasis | 12V | 12V | 233.5 |  | 12V | GnP |
| 5 | f | 69 | Locally advanced | 12R | 12R, 12D | 114.5 |  |  | GnP |
| 6 | f | 76 | Para-aortic LN metastasis | Wild | 12D | 6.8 | 12D | 12D | GnP |
| 7 | f | 44 | Locally advanced | 12D | 12D | 174.9 |  |  | FOLFIRINOX |
| 8 | f | 74 | Liver, lung metastasis | 12R | 12R, 12D | 47.2 |  |  | GnP |
| 9 | f | 81 | LN metastasis | 12V | 12V, 12D | 423.3 |  |  | GnP |
| 10 | m | 65 | Liver metastasis | ND | ND | 62 | 13D | 13D | GnP |
| 11 | m | 63 | Peritoneal dissemination | 12R | 12R, 12D | 14840 | 12R | 12R | FOLFIRINOX |
| 12 | f | 65 | Locally advanced | 12V | 12V | 4.9 | 12V | 12V | GnP |
| 13 | f | 84 | Locally advanced | ND | 12V, 12D | 4223.4 | 12V | 12D, 12V | Gemcitabine |
| 14 | f | 40 | Liver, lung metastasis | ND | 12D | 15276.9 |  | 12D | FOLFIRINOX |
| 15 | m | 72 | Locally advanced | ND | ND | 576 |  | 12D | GnP |
| 16 | f | 74 | Locally advanced | Q61H | Q61H, 12D | 23.1 |  |  | GnP |
| 17 | m | 65 | Liver metastasis | 12V | 12V | 1518 |  | 12V | GnP |
| 18 | f | 72 | Liver metastasis | 12D | 12D | 2024 | 12D | 12D | GnP |
| 19 | m | 70 | Liver metastasis, peritoneal dissemination | 12D | 12D | 1795.4 | 12D | 12D | GnP |
| 20 | m | 68 | Liver, lung metastasis, peritoneal dissemination | 12V | 12V | 32664.5 | 12V | 12V | GnP |
| 21 | m | 69 | Liver metastasis | Q61H | Q61H | 1213.7 | Q61H | Q61H | GnP |
| 22 | m | 72 | Liver, lung metastasis | ND | 12D | 7700 | 12D | 12D | GnP |

ddPCR, droplet digital polymerase chain reaction; CA19-9, carbohydrate antigen 19-9; ctDNA, circulating tumor DNA; LN, lymph node; ND, not determined; NA, not applicable; FOLFIRINOX, folinic acid + fluorouracil + irinotecan + oxaliplatin; GnP, gemcitabine + nab-paclitaxel; blank, no detection of *KRAS*-mutated ctDNA.
