# Supplementary material for: Optimal value of CA19-9 determined by KRAS-mutated circulating tumor DNA contributes to the prediction of prognosis in pancreatic cancer patients
Source: Sci Rep. 2021 Oct 21;11:20797. doi: 10.1038/s41598-021-00060-9 (PMC8531317; doi:10.1038/s41598-021-00060-9)
Supplement: Supplementary file 6 — Supplementary Table S2. [file 41598_2021_60_MOESM6_ESM.docx]

**Supplementary Table S2A.** Univariate and multivariate analyses of overall survival in the surgery group, including patients with jaundice

|  |  | Univariate analysis | | Multivariate analysis | |
| --- | --- | --- | --- | --- | --- |
| Prognostic factors | No. of patients | MST (months) | *P*-value | Hazard ratio (95% CI) | *P*-value |
| Sex |  |  |  |  |  |
| Male | 59 | 25.7 |  |  |  |
| Female | 32 | 33.0 | 0.229 |  |  |
| Age (median, 66.5 years) |  |  |  |  |  |
| ≤67 years | 45 | 29.0 |  |  |  |
| >67 years | 46 | 28.4 | 0.362 |  |  |
| Tumor location |  |  |  |  |  |
| Head | 72 | 21.5 |  | 1 | Reference |
| Body + tail | 19 | 68.4 | 0.005 | 0.431 (0.200-0.926) | 0.031 |
| Tumor size |  |  |  |  |  |
| ≤2cm | 14 | 104.9 |  | 1 | Reference |
| >2cm | 77 | 22.3 | 0.009 | 1.676 (0.626-4.491) | 0.304 |
| T factor (UICC) |  |  |  |  |  |
| T1+T2 | 60 | 33.3 |  | 1 | Reference |
| T3 | 31 | 21.0 | 0.085 | 0.928 (0.536-1.605) | 0.788 |
| Lymph node metastasis |  |  |  |  |  |
| Negative | 25 | 104.9 |  | 1 | Reference |
| Positive | 66 | 22.0 | 0.001 | 2.048 (0.986-4.255) | 0.055 |
| Pathological differentiation |  |  |  |  |  |
| G1+G2 | 81 | 31.7 |  |  |  |
| Others | 10 | 13.8 | 0.151 |  |  |
| CA19-9 level |  |  |  |  |  |
| ≤244.2 U/mL | 45 | 34.0 |  | 1 | Reference |
| >244.2 U/mL | 46 | 21.0 | 0.070 | 0.903 (0.476-1.714) | 0.755 |
| CA19-9 level |  |  |  |  |  |
| ≤949.7 U/mL | 68 | 33.3 |  | 1 | Reference |
| >949.7 U/mL | 23 | 16.1 | 0.002 | 2.111 (1.047-4.258) | 0.037 |
| Adjuvant chemotherapy |  |  |  |  |  |
| No | 21 | 38.9 |  |  |  |
| Yes | 70 | 25.7 | 0.125 |  |  |

MST, median survival time; CI, confidence interval; UICC, Union for International Cancer Control; CA19-9, carbohydrate antigen 19-9.

**Supplementary Table S2B.** Univariate and multivariate analyses of recurrence free survival in the surgery group, including patients with jaundice

|  |  | Univariate analysis | | Multivariate analysis | |
| --- | --- | --- | --- | --- | --- |
| Prognostic factors | No. of patients | MST (months) | *P*-value | Hazard ratio (95% CI) | *P*-value |
| Sex |  |  |  |  |  |
| Male | 59 | 13.1 |  |  |  |
| Female | 32 | 16.4 | 0.152 |  |  |
| Age (median, 66.5 years) |  |  |  |  |  |
| ≤67 years | 45 | 14.8 |  |  |  |
| >67 years | 46 | 13.5 | 0.386 |  |  |
| Tumor location |  |  |  |  |  |
| Head | 72 | 12.8 |  |  |  |
| Body + tail | 19 | 28.1 | 0.120 |  |  |
| Tumor size |  |  |  |  |  |
| ≤2cm | 14 | 38.2 |  | 1 | Reference |
| >2cm | 77 | 12.8 | 0.052 | 1.25 (0.585-2.690) | 0.561 |
| T factor (UICC) |  |  |  |  |  |
| T1+T2 | 60 | 17.0 |  | 1 | Reference |
| T3 | 31 | 9.2 | 0.088 | 1.045 (0.625-1.747) | 0.866 |
| Lymph node metastasis |  |  |  |  |  |
| Negative | 25 | 33.4 |  | 1 | Reference |
| Positive | 66 | 10.7 | 0.004 | 1.792 (0.971-3.304) | 0.062 |
| Pathological differentiation |  |  |  |  |  |
| G1+G2 | 81 | 15.3 |  |  |  |
| Others | 10 | 6.1 | 0.140 |  |  |
| CA19-9 level |  |  |  |  |  |
| ≤221.05 U/mL | 45 | 18.1 |  |  |  |
| >221.05 U/mL | 46 | 10.4 | 0.243 |  |  |
| CA19-9 level |  |  |  |  |  |
| ≤949.7 U/mL | 68 | 16.5 |  | 1 | Reference |
| >949.7 U/mL | 23 | 9.5 | 0.030 | 1.565 (0.911-2.690) | 0.105 |
| Adjuvant chemotherapy |  |  |  |  |  |
| No | 21 | 24.3 |  | 1 | Reference |
| Yes | 70 | 13.2 | 0.052 | 1.545 (0.855-2.790) | 0.149 |

MST, median survival time; CI, confidence interval; UICC, Union for International Cancer Control; CA19-9, carbohydrate antigen 19-9.
