# Supplementary material for: Optimal value of CA19-9 determined by KRAS-mutated circulating tumor DNA contributes to the prediction of prognosis in pancreatic cancer patients
Source: Sci Rep. 2021 Oct 21;11:20797. doi: 10.1038/s41598-021-00060-9 (PMC8531317; doi:10.1038/s41598-021-00060-9)
Supplement: Supplementary file 7 — Supplementary Table S3. [file 41598_2021_60_MOESM7_ESM.docx]

**Supplementary Table S3.** Clinical information of patients who underwent surgery

| No. | Sex | Age | Tumor location | Operation method | Tumor size | T factor | N | stage | Pathological differentiation | Preoperative CA19-9 level | Preoperative Total bilirubin level | Adjuvant chemotherapy | Recurrence | Prognosis |
| --- | --- | --- | --- | --- | --- | --- | --- | --- | --- | --- | --- | --- | --- | --- |
|  |  | (years) |  |  |  | UICC | UICC | UICC |  |  |  |  |  |  |
| 1 | M | 65 | Head | SSPPD | 2.5 | 2 | 1 | IIB | Well | 481 | 1.15 | No | Yes | Alive |
| 2 | M | 54 | Head | SSPPD | 2.5 | 2 | 1 | IIB | Moderately | 245.7 | 0.41 | No | Yes | Death |
| 3 | M | 77 | Head | SSPPD | 3 | 2 | 2 | III | Moderately | 118.7 | 0.99 | Yes | Yes | Death |
| 4 | F | 76 | Body | TP | 2 | 1 | 0 | IA | Moderately | 66.1 | 0.34 | No | Yes | Alive |
| 5 | F | 66 | Head | SSPPD | 2.5 | 2 | 2 | III | Moderately | 224.4 | 8.54 | Yes | No | Death |
| 6 | F | 71 | Head | SSPPD | 1.5 | 1 | 0 | IA | Well | 13.4 | 0.45 | No | No | Alive |
| 7 | M | 79 | Tail | DP | 3.5 | 2 | 1 | IIB | Moderately | 484.2 | 0.44 | No | Yes | Death |
| 8 | M | 64 | Head | SSPPD | 2.5 | 2 | 2 | III | Well | 153.3 | 17.92 | No | Yes | Death |
| 9 | F | 75 | Head | SSPPD | 3 | 2 | 0 | IB | Well | 312.2 | 6.97 | Yes | Yes | Death |
| 10 | M | 76 | Body | TP | 5 | 3 | 1 | IIB | Moderately | 654 | 1.76 | Yes | No | Death |
| 11 | M | 67 | Head | SSPPD | 2.6 | 2 | 1 | IIB | Moderately | 339.7 | 1.11 | Yes | Yes | Death |
| 12 | M | 63 | Head | SSPPD | NA | 3 | 1 | IIB | Moderately | 2 | 6 | Yes | Yes | Death |
| 13 | M | 65 | Head | SSPPD | 3 | 2 | 2 | III | Well | 2 | 0.42 | Yes | Yes | Death |
| 14 | F | 66 | Head | TP | 7 | 3 | 1 | IIB | Well | 2 | 0.62 | No | Yes | Death |
| 15 | F | 56 | Body | DP | 3.8 | 2 | 1 | IIB | Well | 3149 | 0.58 | Yes | Yes | Death |
| 16 | M | 70 | Body | DP | 4 | 3 | 1 | IIB | Moderately | 1153 | 0.61 | Yes | Yes | Alive |
| 17 | M | 77 | Head | SSPPD | 2 | 1 | 2 | III | Moderately | 43.6 | 1.07 | No | No | Death |
| 18 | M | 62 | Head | SSPPD | 4 | 3 | 1 | IIB | Well | 176.5 | 1.11 | No | Yes | Alive |
| 19 | F | 46 | Head | SSPPD | 2.6 | 2 | 1 | IIB | Well | 136.1 | 1.41 | Yes | Yes | Death |
| 20 | M | 70 | Head | SSPPD | 1.8 | 1 | 1 | IIB | Poorly | 855 | 9.88 | No | Yes | Death |
| 21 | M | 63 | Head | SSPPD | 3.5 | 2 | 1 | IIB | Moderately | 130.4 | 12.08 | Yes | Yes | Death |
| 22 | F | 71 | Head | SSPPD | 4 | 3 | 2 | III | Well | 21340 | 4.39 | Yes | Yes | Death |
| 23 | M | 62 | Head | SSPPD | 3.5 | 2 | 1 | IIB | Moderately | 94.4 | 8.75 | Yes | Yes | Death |
| 24 | M | 74 | Head | SSPPD | 3.1 | 2 | 1 | IIB | Well | 774 | 8.19 | Yes | Yes | Death |
| 25 | M | 40 | Head | SSPPD | 3 | 2 | 1 | IIB | Well | 67.5 | 12.86 | Yes | Yes | Death |
| 26 | M | 66 | Head | SSPPD | 4.5 | 3 | 2 | III | Well | 224.8 | 2.21 | No | Yes | Death |
| 27 | F | 58 | Tail | DP | 3.1 | 2 | 1 | IIB | Well | 1292 | 0.44 | Yes | No | Alive |
| 28 | M | 55 | Head | SSPPD | 4 | 3 | 2 | III | Poorly | 63.1 | 8.99 | Yes | Yes | Death |
| 29 | M | 67 | Body | DP | 5 | 3 | 1 | IIB | Moderately | 608 | 0.15 | Yes | Yes | Alive |
| 30 | M | 57 | Head | SSPPD | 4 | 3 | 1 | IIB | Well | 477.4 | 0.89 | Yes | No | Alive |
| 31 | F | 71 | Head | SSPPD | 3.5 | 2 | 2 | III | Well | 684 | 2.28 | No | Yes | Death |
| 32 | M | 37 | Head | SSPPD | 4 | 3 | 2 | III | Poorly | 1421 | 4.62 | Yes | NA | Death |
| 33 | M | 59 | Head | SSPPD | 2.5 | 2 | 1 | IIB | Well | 50.6 | 7.09 | Yes | Yes | Death |
| 34 | F | 73 | Tail | DP | 7.5 | 3 | 1 | IIB | Papillary | 28840 | 0.92 | Yes | Yes | Death |
| 35 | M | 71 | Head | SSPPD | 4.1 | 3 | 1 | IIB | Well | 274.1 | 1.34 | Yes | Yes | Death |
| 36 | F | 61 | Head | SSPPD | 3 | 2 | 1 | IIB | Well | 244.2 | 6.05 | No | No | Alive |
| 37 | F | 49 | Head | SSPPD | 1.6 | 1 | 0 | IA | Poorly | 8.6 | 0.52 | No | No | Alive |
| 38 | F | 66 | Head | SSPPD | 1.7 | 1 | 0 | IA | Moderately | 20.2 | 0.34 | Yes | Yes | Death |
| 39 | M | 77 | Head | SSPPD | 4 | 3 | 0 | IIA | Moderately | 7840 | 1.2 | Yes | Yes | Death |
| 40 | M | 53 | Head | SSPPD | 3.5 | 2 | 1 | IIB | Well | 286.3 | 3.23 | Yes | No | Death |
| 41 | M | 65 | Body | TP | 4.2 | 3 | 1 | IIB | Well | 382 | 0.7 | No | No | Alive |
| 42 | M | 68 | Head | SSPPD | 4.5 | 3 | 2 | III | Well | 3908 | 26.94 | No | Yes | Death |
| 43 | F | 68 | Head | SSPPD | 3.8 | 2 | 2 | III | Well | 968 | 7.31 | Yes | No | Alive |
| 44 | M | 58 | Head | SSPPD | 3.8 | 2 | 1 | IIB | Well | 162.5 | 0.82 | Yes | Yes | Death |
| 45 | M | 65 | Head | SSPPD | 4.4 | 3 | 1 | IIB | Well | 211.3 | 14.69 | Yes | Yes | Death |
| 46 | M | 68 | Body | DP | 1.7 | 1 | 0 | IA | Well | 51.7 | 0.48 | Yes | Yes | Death |
| 47 | M | 67 | Head | SSPPD | 2.6 | 2 | 0 | IB | Moderately | 204.6 | 13.56 | Yes | Yes | Death |
| 48 | F | 77 | Head | SSPPD | 3.5 | 2 | 0 | IB | Well | 2223 | 16 | Yes | Yes | Death |
| 49 | M | 64 | Head | SSPPD | 3.8 | 2 | 2 | III | Moderately | 273.2 | 3.39 | Yes | Yes | Death |
| 50 | M | 65 | Body | DP | 1.5 | 1 | 1 | IIB | Well | 13.4 | 0.43 | Yes | Yes | Alive |
| 51 | M | 67 | Head | SSPPD | 3.5 | 2 | 0 | IB | Well | 59.6 | 7.58 | Yes | Yes | Death |
| 52 | M | 75 | Head | SSPPD | 4.2 | 3 | 1 | IIB | Well | 1087 | 6.31 | Yes | Yes | Death |
| 53 | M | 70 | Head | SSPPD | 2 | 1 | 0 | IA | Well | 44.9 | 4.97 | Yes | Yes | Death |
| 54 | M | 61 | Head | SSPPD | 4.3 | 3 | 2 | III | Well | 4271 | 12.47 | Yes | No | Death |
| 55 | M | 52 | Body | TP | 4.8 | 3 | 0 | IIA | Well | 2 | 0.72 | Yes | No | Death |
| 56 | F | 56 | Body | DP | 3.3 | 2 | 1 | IIB | Well | 162.5 | 0.43 | No | Yes | Death |
| 57 | F | 72 | Head | SSPPD | 2.5 | 2 | 1 | IIB | Moderately | 637 | 0.78 | Yes | Yes | Death |
| 58 | F | 66 | Head | SSPPD | 4.2 | 3 | 1 | IIB | Well | 36.3 | 1.09 | Yes | Yes | Death |
| 59 | M | 73 | Head | SSPPD | 5 | 3 | 1 | IIB | Well | 2139 | 0.25 | Yes | No | Death |
| 60 | M | 72 | Head | SSPPD | 3.8 | 2 | 0 | IB | Well | 24640 | 2.52 | Yes | Yes | Death |
| 61 | M | 73 | Head | SSPPD | 3.8 | 2 | 2 | III | Well | 2 | 19.12 | Yes | Yes | Death |
| 62 | M | 55 | Body | DP | 3.5 | 2 | 1 | IIB | Well | 936 | 1.07 | Yes | Yes | Death |
| 63 | M | 55 | Head | SSPPD | 5.7 | 3 | 2 | III | Well | 275.2 | 0.82 | Yes | Yes | Death |
| 64 | M | 66 | Head | SSPPD | 3.8 | 2 | 0 | IB | Well | 2 | 1.59 | Yes | No | Death |
| 65 | M | 52 | Body | DP | 2.6 | 2 | 1 | IIB | Well | 759 | 1.45 | Yes | No | Alive |
| 66 | M | 62 | Head | SSPPD | 3 | 2 | 1 | IIB | Well | 217.3 | 0.98 | Yes | Yes | Death |
| 67 | F | 66 | Body | DP | 3.2 | 2 | 0 | IB | Adenosquamous | 55.4 | 1.21 | Yes | Yes | Alive |
| 68 | F | 41 | Head | SSPPD | 2.4 | 2 | 1 | IIB | Well | 162 | 13.18 | Yes | Yes | Alive |
| 69 | F | 52 | Head | SSPPD | 3.8 | 2 | 2 | III | Well | 26.5 | 1.64 | Yes | Yes | Death |
| 70 | F | 67 | Head | SSPPD | 7.8 | 3 | 1 | IIB | Well | 2 | 2.13 | Yes | Yes | Death |
| 71 | F | 70 | Head | SSPPD | 3.7 | 2 | 2 | III | Well | 2 | 6.16 | Yes | Yes | Death |
| 72 | M | 73 | Head | SSPPD | 3.7 | 2 | 0 | IB | Well | 88.4 | 4.82 | Yes | Yes | Death |
| 73 | M | 70 | Head | SSPPD | 3.8 | 2 | 1 | IIB | Well | 1746 | 21.99 | Yes | Yes | Death |
| 74 | M | 65 | Body | DP | 2.3 | 2 | 1 | IIB | Well | 11.8 | 0.4 | Yes | No | Alive |
| 75 | M | 78 | Head | SSPPD | 5 | 3 | 1 | IIB | Papillary | 3324 | 0.5 | Yes | Yes | Death |
| 76 | F | 60 | Head | SSPPD | 4.4 | 3 | 2 | III | Poorly | 1165 | 11.12 | Yes | No | Death |
| 77 | M | 71 | Head | SSPPD | 1.5 | 1 | 0 | IA | Well | 117.7 | 0.59 | Yes | Yes | Alive |
| 78 | M | 70 | Head | SSPPD | 2.4 | 2 | 0 | IB | Well | 187 | 2.45 | Yes | Yes | Death |
| 79 | F | 74 | Head | SSPPD | 3.5 | 2 | 2 | III | Well | 138.3 | 1.33 | Yes | No | Death |
| 80 | M | 78 | Head | SSPPD | 3.7 | 2 | 1 | IIB | Well | 57.9 | 0.45 | Yes | Yes | Death |
| 81 | M | 39 | Head | SSPPD | 3 | 2 | 0 | IB | Well | 13 | 1 | No | No | Alive |
| 82 | M | 61 | Head | SSPPD | 5.2 | 3 | 1 | IIB | Well | 240.2 | 0.5 | Yes | No | Alive |
| 83 | F | 78 | Body | DP | 2.7 | 2 | 0 | IB | Well | 465.3 | 0.99 | No | No | Alive |
| 84 | M | 71 | Head | SSPPD | 5.5 | 3 | 2 | III | Moderately | 39 | 1.06 | Yes | Yes | Death |
| 85 | F | 65 | Head | SSPPD | 4 | 3 | 0 | IIA | Well | 85 | 1.84 | No | No | Alive |
| 86 | F | 74 | Body | DP | 2 | 1 | 0 | IA | Well | 1013 | 0.35 | No | No | Alive |
| 87 | M | 44 | Tail | DP | 1.5 | 1 | 0 | IA | Well | 132.4 | 0.57 | Yes | Yes | Alive |
| 88 | F | 73 | Head | SSPPD | 3.8 | 2 | 2 | III | Moderately | 482.1 | 12.01 | Yes | Yes | Death |
| 89 | F | 73 | Head | SSPPD | 2.6 | 2 | 1 | IIB | Papillary | 71.8 | 0.59 | Yes | Yes | Death |
| 90 | F | 70 | Tail | DP | 4.5 | 3 | 0 | IIA | Well | 55.4 | 0.42 | Yes | Yes | Alive |
| 91 | F | 72 | Head | SSPPD | 3 | 2 | 0 | IB | Well | 374.4 | 11.48 | Yes | No | Alive |
| 92 | M | 68 | Head | SSPPD | 2.3 | 2 | 0 | IB | Poorly | 2 | 2.01 | No | No | Death |
| 93 | M | 71 | Head | SSPPD | 3.5 | 2 | 2 | III | Well | 12800 | 1.12 | Yes | Yes | Death |
| 94 | F | 58 | Tail | DP | 3.7 | 2 | 0 | IB | Well | 2452 | 0.78 | Yes | Yes | Death |
| 95 | F | 63 | Head | SSPPD | 4.5 | 3 | 2 | III | Moderately | 16.1 | 0.38 | Yes | Yes | Death |
| 96 | M | 69 | Head | SSPPD | 3.6 | 2 | 1 | IIB | Well | 244.6 | 6.22 | No | No | Death |
| 97 | F | 63 | Head | SSPPD | 2.5 | 2 | 0 | IB | Well | 2 | 0.6 | No | No | Alive |
| 98 | F | 61 | Head | SSPPD | 4.5 | 3 | 1 | IIB | Anaplastic | 107.4 | 0.51 | Yes | Yes | Death |
| 99 | M | 52 | Head | SSPPD | 4.9 | 3 | 1 | IIB | Well | 3507 | 1.26 | Yes | No | Death |
| 100 | M | 75 | Head | SSPPD | 6.5 | 3 | 1 | IIB | Well | 2946 | 0.62 | No | No | Death |
| 101 | F | 70 | Head | TP | 2.5 | 2 | 1 | IIB | Well | 932 | 0.35 | No | No | Alive |
| 102 | M | 63 | Head | SSPPD | 4.2 | 3 | 1 | IIB | Tub | 8840 | 0.50 | Yes | Yes | Death |
| 103 | M | 79 | Head | SSPPD | 2 | 1 | 0 | IA | Well | 56.5 | 0.47 | Yes | No | Alive |
| 104 | M | 44 | Head | SSPPD | 4 | 3 | 1 | IIB | Well | 1080 | 2.75 | Yes | Yes | Death |

SSPPD, subtotal stomach-preserving pancreaticoduodenectomy; DP, distal pancreatectomy; TP, total pancreatectomy; UICC, Union for International Cancer Control; CA19-9, carbohydrate antigen 19-9; NA, not applicable
